# Improvement of Starch Digestion Using *α*-Amylase Entrapped in Pectin-Polyvinyl Alcohol Blend

**DOI:** 10.1155/2015/145903

**Published:** 2015-04-08

**Authors:** Maurício Cruz, Kátia Fernandes, Cristine Cysneiros, Reginaldo Nassar, Samantha Caramori

**Affiliations:** ^1^Departamento de Química, Instituto Federal de Educação Ciência e Tecnologia de Goiás, 76400-000 Uruaçu, GO, Brazil; ^2^Departamento de Bioquímica e Biologia Molecular, Laboratório de Química de Polímeros, Universidade Federal de Goiás, Caixa Postal 131, 74001-970 Goiânia, GO, Brazil; ^3^Departamento de Ciências Fisiológicas, Laboratório de Fisiologia da Digestão, Universidade Federal de Goiás, Caixa Postal 131, 74001-970 Goiânia, GO, Brazil; ^4^Laboratório de Biotecnologia, Unidade Universitária de Ciências Exatas e Tecnológicas, Universidade Estadual de Goiás, 75.132-903 Anápolis, GO, Brazil

## Abstract

Polyvinyl alcohol (PVA) and pectin blends were used to entrap *α*-amylase (Termamyl) using glutaraldehyde as a cross-linker. The effect of glutaraldehyde concentration (0.25, 0.5, 0.75, 1.0, and 1.25%) on the activity of the immobilized enzyme and rate of enzyme released was tested during a 24 h period. Characteristics of the material, such as scanning electron microscopy (SEM), tensile strength (TS), elongation, and rate of dissolution in water (pH 5.7), ruminal buffering solution (pH 7.0), and reactor containing 0.1 mol L^−1^ sodium phosphate buffer (pH 6.5), were also analyzed. SEM results showed that the surfaces of the pectin/PVA/amylase films were highly irregular and rough. TS values increased as a function of glutaraldehyde concentration, whereas percentage of elongation (%*E*) decreased. Pectin/PVA/amylase films presented similar values of solubility in the tested solvents. The material obtained with 0.25% glutaraldehyde performed best with repeated use (active for 24 h), in a phosphate buffer reactor. By contrast, the material obtained with 1.25% glutaraldehyde presented higher performance during *in vitro* testing using an artificial rumen. The results suggest that pectin/PVA/amylase is a highly promising material for biotechnological applications.

## 1. Introduction

*α*-Amylases (*α*-1,4-glucan-4-glucanohydrolases; E.C. 3.2.1.1) belong to an important class of digestive enzymes that catalyze the hydrolysis of *α*-D-(1,4)-glucan linkages present in starch [1, 2]. They play an essential role in the digestive processes of animals, since they provide low molecular weight carbohydrates (dextrins). Amylases are also responsible for the hydrolysis of polysaccharides presented in diets rich in carbohydrates, including those used in cattle management [3].

Although carbohydrate-rich diets contribute to higher energy gain in these animals, scientific studies have shown that a large amount of undigested starch can be measured in their feces, implying a low efficiency, if not inefficiency, of the digestive enzymes in corn processing [3, 4]. The content of *α*-amylase in the digestive tract of ruminants is crucial for their use of the energy content of their diets [4, 5]. Thus, a mechanism that enables a gradual increase of amylolytic activity in rumen contents could aid ruminants' digestion of polysaccharides.

In this context, the entrapment of enzymes in a soluble matrix appears as a tool for allowing the continuous release of the enzyme in the reaction medium. There is a wide availability of polymer matrices that can be used for enzyme entrapment, including alginate, chitosan, cashew gum, polyvinyl alcohol (PVA), and pectin [6–8]. In fact, several authors have reported using entrapped *α*-amylase for starch hydrolysis [9, 10]. Entrapment is frequently obtained via a cross-linking reaction. The cross-linking process occurs with the formation of cross-binding between the linker molecule and polymer chains. The linker molecule generally has lower molecular weight than that of the backbone and at least two reactive functional groups in its structure [11]. Glutaraldehyde is considered to be an excellent cross-linking agent in the formation of networks due to the reactivity of the aldehyde groups located at their ends [12, 13].

In a recent study, the fruit of the lobeira, plant from Brazilian Cerrado, has been noted as an excellent source of pectin due to its high productivity as well as its few applications as food [14, 15]. Nevertheless, the lobeira is a common and abundant plant resistant to hydric and climatic stress that survives and fructifies throughout the year with high productivity, all of which makes the plant attractive for biotechnological exploitation [14].

Pectin is a polysaccharide constituted by units of D-galacturonic acid linked by bonds (*α*-1,4) with branching points composed of residues of rhamnose and arabinose. Pectin has been applied in the synthesis of polymeric matrices for drug-controlled release, the entrapment of proteins and cells [16, 17]. Pectin is classified among soluble fibers, which are resistant to hydrolysis from digestive enzymes, yet broken into small fragments by enzymes produced by ruminal microorganisms. This characteristic enables pectin to be applied as an interesting alternative for ruminal delivery system, since this natural polymer remains intact until reaching the ruminal environment.

In this sense, the objective of the present work was to synthetize a polymeric matrix for the slow ruminal release of *α*-amylase by blending polyvinyl alcohol (PVA) with pectin from lobeira fruit, using glutaraldehyde as the cross-linking agent. This matrix was used to entrap *α*-amylase aiming to use the material as an additive to improve the digestion of polysaccharides in an artificial rumen.

## 2. Materials and Methods

### 2.1. Materials

#### 2.1.1. *α*-Amylase (E.C 3.2.1.1)

The commercial thermostable *α*-amylase Termamyl produced by Novozymes Latin America Ltda. (Araucária, Brazil) was used for immobilization procedures. The *α*-amylase solution was prepared by diluting the stock enzyme solution 1 : 10 (v/v) in distilled water, which resulted in an activity of 413 U mL^−1^ (±6.7) and an average specific activity of 256 U mg^−1^ of protein. One enzyme unit was defined as the amount of *α*-amylase that produces 1.0 *μ*mol of reducing sugar/mL/min.

#### 2.1.2. Pectin of Lobeira (*Solanum lycocarpum* St. Hil)

The lobeira fruits used in this study were collected in Goiânia, Goiás, Brazil (16° 35′ 56.0′′ S, 49° 16′ 49.0′′ W), and Anápolis, Goiás, Brazil (16° 22′ 53.1′′ S, 48° 56′ 49.4′′ W).

Two collections during February and March 2012 were performed. The extraction protocol for pectin employed the method described by Torralbo et al. with a yield of approximately 33% [14]. Briefly, flour from dry pulp was suspended in water at a ratio of 1 g/30 mL. The pH was adjusted to 1.0 with 70% (v/v) nitric acid solution, after which the mixture was heated to 80°C and the extraction carried out with continuous stirring for 30 min. The hot acid extract was vacuum-filtered, cooled to 4°C, and precipitated with two volumes of cold ethanol. The isolated pectin pellet was washed twice with cold ethanol, and the solid material was dried at 60°C in an air-forced oven.

### 2.2. Preparation of the Pectin/PVA/Amylase Film

The synthesis of pectin/PVA/amylase blends was performed by varying the concentration of the cross-linking agent glutaraldehyde. Briefly, 2.4 g of lobeira pectin and 0.24 g of PVA were added to 20 mL of distilled water (Vetec Química Fina Ltda.). The solutions were kept stirred at room temperature (25°C), and, after homogenization, 2 mL of *α*-amylase (as in Section 2.1.1) was added. Thereafter, different volumes of 25% (v/v) glutaraldehyde (Vetec Química Fina Ltda.) were added to final concentrations of 0.25, 0.5, 0.75, 1.0, and 1.25% (v/v). The system was maintained with stirring for 10 min at 25°C. The resulting solutions were deposited on molds, and films were produced by casting. After being removed from the molds, the films were milled in a knife mill (Tecnal, Brazil) to obtain grids with an average size of 500 *μ*m.

### 2.3. Mechanical Properties

Mechanical properties were determined in a texture analyzer (TA.TX2, Stable Micro Systems, Surrey, UK), with a 50 N load cell equipped with tensile grips (A/TG model). Samples of the pectin/PVA/amylase film were cut into strips of 20 mm × 40 mm, according to the ASTM D-638M-93 standard [18]. The grip separation was set at 25 mm with a cross-head speed of 500 mm min^−1^. Tensile strength (TS) and percentage of elongation (%*E*) were evaluated. Each sample used was previously inspected, and those containing any artifact such as air bubbles, holes, and tears or showing average thickness variation higher than 5% were rejected.

### 2.4. Surface Characterization of Pectin/PVA/Amylase

The surface characterization of pectin/PVA/amylase was performed in a scanning electron microscope (JEOL, JSM 6610, USA) at the Laboratory of Electron Microscopy in the Institute of Physics at Universidade Federal de Goiás. The dried samples were placed in the holder of four cavities and visualized with an increase of 5.0 microns.

### 2.5. Solubility Tests

The solubility of pectin/PVA/amylase films in water and rumen buffer was determined according to Gontard et al. [19]. Samples were heated at 105°C for 24 h to determine the initial water content. Thereafter, 500 mg of the dried grids was immersed in 50 mL of distilled water or rumen buffer and left standing at 20°C for 24 h. The mixtures were filtered, and materials retained in the filters were dried at 105°C for 24 h. The solubility of the films was expressed as the percentage of soluble mass in relation to the total mass according the following equation:

(1) $$\begin{array}{c} \text{Solubility}\;\;\left(\%\right)=\left[\frac{\left(W_{i}-W_{f}\right)}{W_{i}}\right]\times 100, \end{array}$$

in which *W*_*i*_ is the initial weight of the sample (mg) and *W*_*f*_ is the weight of the dried material retained in the filter (mg).

### 2.6. Assay of Immobilized *α*-Amylase in Pectin/PVA/Amylase Films

The amylolytic activity of free *α*-amylase was determined by the formation of reducing sugars from the starch solution 0.5% (w/v) in the presence of 3,5-dinitrosalicylic acid (ADNS) as described previously by Bernfeld [20], with modifications. The reaction system was composed of 10 mg of the grids of pectin/PVA/amylase in 100 *μ*L of 0.1 mol L^−1^ sodium phosphate buffer pH 6.5 and 100 *μ*L of 0.5% (w/v) starch solution. The system was maintained with stirring at 40°C for 15 min. Subsequently, an aliquot of 100 *μ*L was withdrawn and added to 900 *μ*L of ADNS. This system was maintained for 5 min in a water bath at 100°C, and, after reaching room temperature (~25°C), a reading was performed at 550 nm (UV Bel Photonics 2000). One unit (U) of *α*-amylase was defined as the amount of enzyme that produced 1.0 *μ*mol of reducing sugar per 1 min of reaction. Assays of the blank and control groups included all reagents except the grids and starch, which were not present in the blank and control groups, respectively.

### 2.7. Determination of Specific Activity of Immobilized *α*-Amylase

The specific activity of pectin/PVA/amylase was determined by washing the grids for 30 min with 0.5 mL of 0.1 mol L^−1^ sodium phosphate buffer pH 6.5. The material was centrifuged at 5000 g, and *α*-amylase activity and protein content in the supernatant were determined. Total protein content was calculated using the method of Qubit (Invitrogen, USA), which is based on a high sensitivity fluorescent reaction between protein and a fluorophore compound from the Qubit assay kit. The specific activity was obtained by solving the following equation:

(2) $$\begin{array}{c} \text{SA}=\frac{E_{\left(\text{immobilized}\right)}}{P_{\left(\text{immobilized}\right)}}=\frac{E_{\left(\text{free}\right)}-E_{\left(\text{washed}\right)}}{P_{\left(\text{free}\right)}-P_{\left(\text{washed}\right)}}, \end{array}$$

in which SA is the specific activity of pectin/PVA/amylase (U mg^−1^ of protein); *E*_(immobilized)_ is the activity of the entrapped amylase (U mL^−1^); *P*_(immobilized)_ is the amount of protein entrapped in the matrix (mg mL^−1^); *E*_(free)_ is the amount of amylase (U mL^−1^) offered for entrapment in the matrix; *E*_(washed)_ is the amylase activity (U mL^−1^) quantified in the supernatant; *P*_(free)_ is the protein concentration (mg mL^−1^) offered for entrapment in the matrix; and *P*_(washed)_ is the protein concentration (mg mL^−1^) quantified in the supernatant.

### 2.8. Monitoring the Rate of Enzyme Release

#### 2.8.1. Enzymatic Reactor Containing Sodium Phosphate Buffer

To examine the stability of the pectin/PVA/amylase, tests of repeated use for starch hydrolysis were conducted in a reactor containing 0.1 mol L^−1^ phosphate buffer pH 6.5, under constant stirring. In these tests, 20 mg of pectin/PVA/amylase grids was incubated with starch solution (as in Section 2.6), and the rate of hydrolysis was monitored every 2 h during a period of 24 h. For all assays, *α*-amylase activity was discounted from the blanks and controls. At the end of the experiment, the remaining pectin/PVA/amylase grids were dried and subsequently weighed for purposes of calculating the percentage of solubility according to (1).

#### 2.8.2. Enzymatic Reactor Containing Ruminal Buffer

The blends produced with concentrations of glutaraldehyde of 0.25% and 1.25% were evaluated for their ability to continuously hydrolyze starch in an artificial rumen of cattle (*in vitro*). For this, some reactors were prepared with their compositions, as detailed in Table 1.

The particles of corn grains used in the reactors had diameters smaller than 500 *μ*m. After the incubation of the blends in the reactors, three aliquots of 1.0 mL were collected to quantify the content of reducing sugar (time zero), and subsequent measurements were taken every 2 h during an incubation period of 12 h, after which the quantification was performed every 12 h during an incubation period of 48 h.

## 3. Results and Discussion

Several studies have verified the possibility of increasing enzyme stability through multiple covalent attachments of their subunits to a matrix support [21]. The process of cross-linking with glutaraldehyde presents several advantages, among which is the fact that the method is quite simple and highly efficient, and poses the possibility to be associated with a wide variety of natural and synthetic supports, including alginate, chitosan, gelatin, nylon, and silica [22, 23].

**Figure 1:**
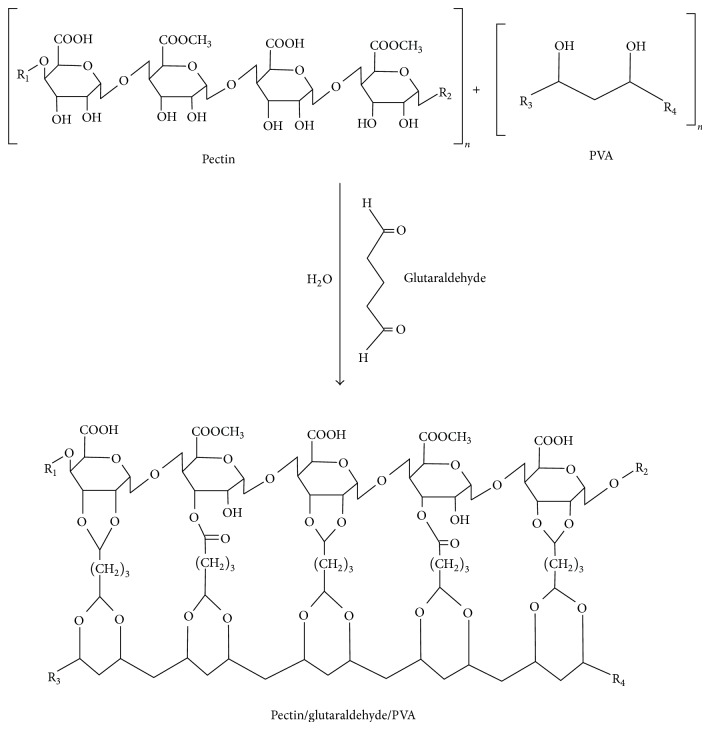
Proposed chemical structure of the pectin/glutaraldehyde/PVA blend.

The structure of the pectin/PVA/amylase matrix cross-linked with glutaraldehyde may have been obtained via the formation of covalent bonds between the carbon 3 of the D-galacturonic acid residue present in the structure of pectin and the carbonyl group located in one end of the glutaraldehyde; the other free end of the glutaraldehyde molecule binds at two hydroxyl groups present in the polymeric chain of the PVA (Figure 1). Pectin has a highly complex structure, which challenges most researchers' representations of all information available in a single structural model. Therefore, the structure proposed in this study considers the predominance of D-galacturonic acid residues, which represent approximately 60–65% of the total pectin molecule [24]. For instance, the existence of other linkages in the proposed model is possible due to chemical interactions between glutaraldehyde and other moieties of pectin, including the residues of D-rhamnose and D-arabinose [25].

### 3.1. Mechanical Properties

TS is defined as the capacity of resistance to rupture, as presented by material when submitted to pressure force [26]. The results obtained in tests of TS and %*E* appear in Table 2. As shown, the measurements of TS showed an increasing trend toward an increased concentration of the cross-linking agent glutaraldehyde. The pectin/PVA/amylase film produced with 1.25% of glutaraldehyde solution showed higher TS of about 126.4% than the material produced using 0.25% of glutaraldehyde. By contrast, the pectin/PVA/amylase film produced with 0.25% of glutaraldehyde presented %*E* 58.4% higher than the 1.25% glutaraldehyde film. Increasing the concentration of the cross-linking agent resulted in the higher availability of molecules to form linkages with the polymeric chains of PVA and pectin, as the reaction model proposed for the cross-linking process shows in Figure 1. The TS of pectin/PVA/amylase films was lower than that reported for commercial plastics such as PVA [27], polypropylenes [28], and polyethylene [29]. Similarly, the values of TS for pectin/PVA/amylase films were lower than those reported for other films containing pectin, such as amidated pectin/alginate [30], irradiated CaCl_2_/citric pectin [31], and apple pectin/alginate [32]. At the same time, the values of %*E* for pectin/PVA/amylase were higher than those reported in literature addressing pectin [30–32].

The properties of resistance of the films can be modified by increasing their thickness. Due to their versatility, films can be made to meet specific goals aimed at different applications, in both industrial and biotechnological scenarios.

### 3.2. Appearance of the Films

**Figure 2:**
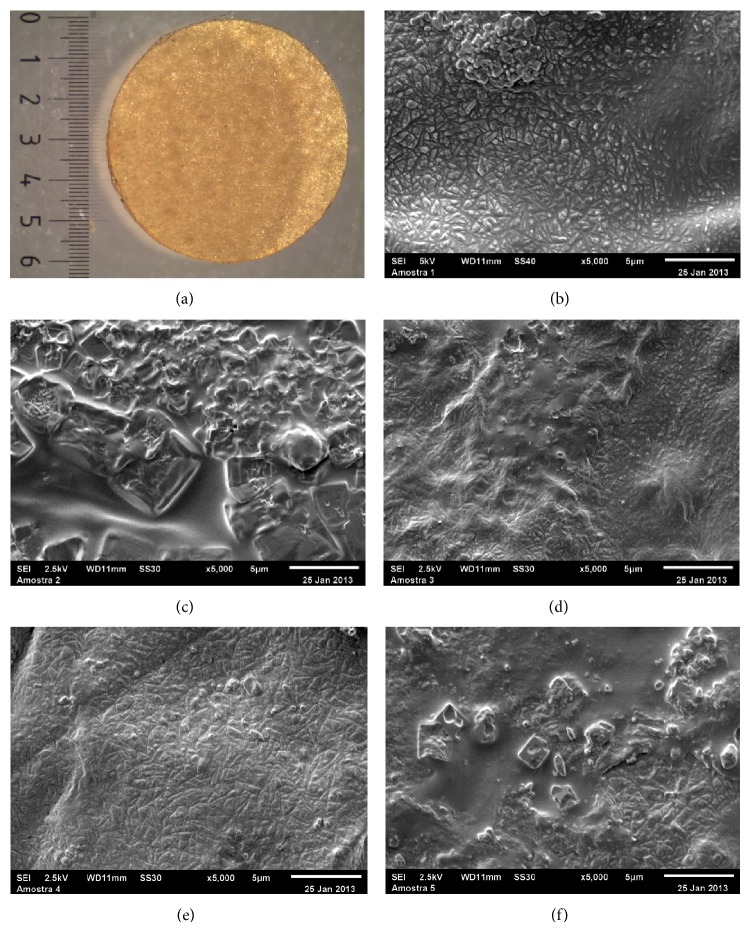
Photographs and scanning electron microscopy (SEM) of pectin/PVA/amylase films. (a) Pectin/PVA/amylase film; ((b)–(f)) SEM pectin/PVA/amylase film produced with 0.25%, 0.5%, 0.75%, 1.00%, and 1.25% glutaraldehyde, respectively.

The produced films presented no fractures or breaks, high flexibility, and easy handling, and their coloration was uniform (Figure 2(a)). The SEM of the films showed that the materials have a highly irregular, rough surface (Figures 2(b), 2(c), 2(d), 2(e), and 2(f)). The films containing glutaraldehyde in concentrations of 0.25%, 0.75%, and 1.0% had a rough surface of a more homogeneous appearance than the film produced in a concentration of 1.25% glutaraldehyde. It seems that lower concentrations of glutaraldehyde result in a network of pectin/PVA/amylase with several chains partially linked to each other, thereby conferring roughness to the material. In concentrations of more than 1.0%, all chains were linked to each other, which resulted in smoother surfaces.

### 3.3. Tests of Solubility

**Figure 3:**
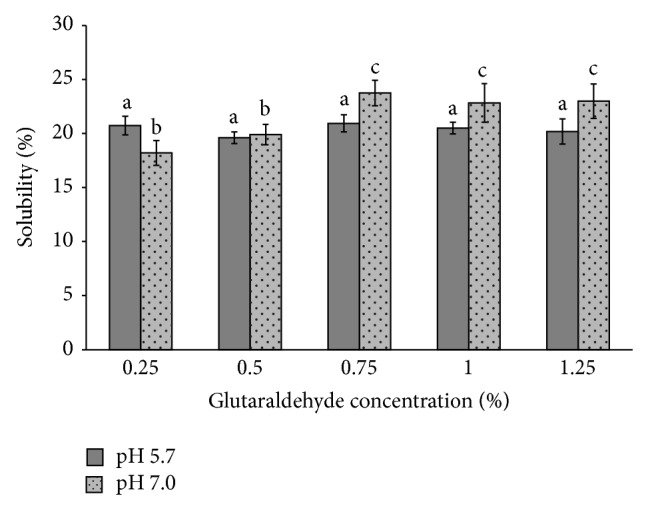
Solubility of pectin/PVA/amylase in different pH conditions. Different letters on the bars indicate statistically significant differences (*p* < 0.05).

The grids of pectin/PVA/amylase showed similar solubility in water (pH 5.7) and ruminal buffer (pH 7.0) with values of approximately 20%, as shown in Figure 3.

Water solubility is related to the content of free hydroxyl groups in the polymeric matrix, which allows the formation of hydrogen interactions between film and water molecules [33]. High solubility values are a desirable characteristic for biodegradable films, since the increase in solubility leads to increased biodegradability and thus enzyme release [34]. The solubility demonstrated by the films, therefore, indicates that the feasible materials pose promising potential for applications in different branches of biotechnology.

### 3.4. Assay of Immobilized *α*-Amylase and Specific Activity

**Figure 4:**
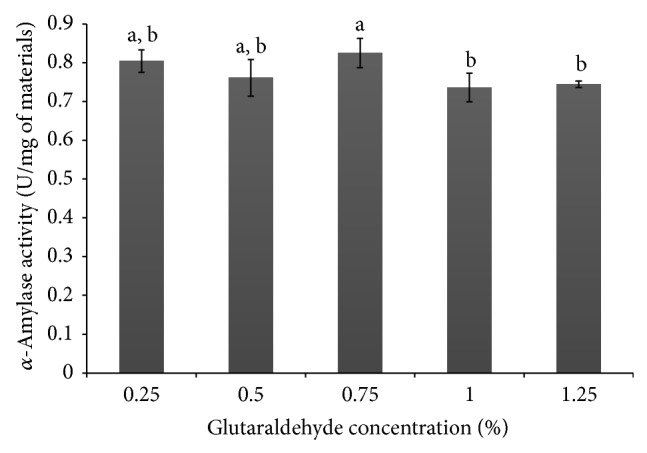
The *α*-amylase activity in the grids of pectin/PVA/amylase produced with different concentrations of glutaraldehyde.

**Figure 5:**
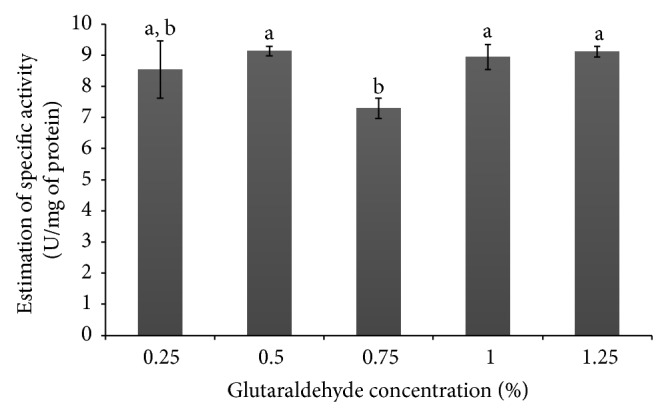
Estimation of specific activity in the grids of pectin/PVA/amylase produced with different concentrations of glutaraldehyde.

The data presented in Figure 4 show that the variation in the concentration of glutaraldehyde did not significantly affect the activity of *α*-amylase (*p* < 0.05) in the materials. The specific activity obtained in the grids of pectin/PVA/amylase (Figure 5) pointed to increased activity at concentrations of 0.5%, 1.0%, and 1.25% glutaraldehyde. However, the difference among values was not significant when tested statistically (*p* < 0.05). The fact that the concentration of glutaraldehyde did not interfere in *α*-amylase activity indicates that the network of pectin/PVA/amylase provides the necessary enzyme access to the polymeric substrate (starch) to produce a similar rate of hydrolysis. Although the cages produced to hold the entrapped enzyme molecule did not restrain enzyme activity, increasing the concentration of glutaraldehyde interfered in the mechanical properties of the resulting material, thereby conferring stability to the network and enzyme support under reaction conditions. At the same time, the pectin/PVA/amylase matrix showed very good solubility (Section 3.3), thus assuring the continuous release of *α*-amylase in the reaction medium.

### 3.5. Monitoring the Rate of Enzymatic Activity

#### 3.5.1. Enzymatic Reactor Containing Sodium Phosphate Buffer

The rate of hydrolysis in the reaction medium is greatly influenced by the concentration of glutaraldehyde used to produce the grids (Table 3). Grids produced with the glutaraldehyde concentration of 0.25% presented the best performance, which allowed the repeated use of amylase over 24 h. The grids containing 0.5% glutaraldehyde preserved enzyme activity for 10 h of incubation, and grids produced with 0.75% and 1.0% glutaraldehyde released enzyme for 8 h and 6 h of incubation, respectively.

An additional observation was the high rate of solubility obtained in this reactor, which reached 84.3% after 24 h of reaction. This result suggests that stirring and the continuous renovation of the reaction solution enhanced the rate of dissolution of pectin/PVA/amylase to a great extent.

#### 3.5.2. Enzymatic Reactor Containing Ruminal Liquid

**Figure 6:**
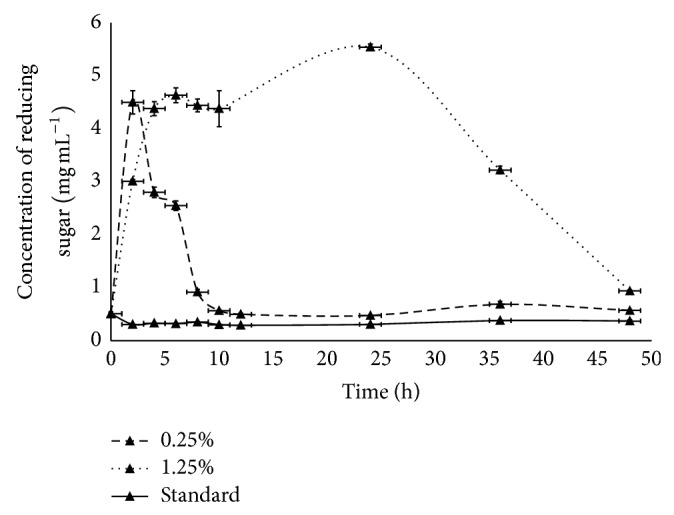
Hydrolytic potential of pectin/PVA/amylase blends over 48 h of incubation in the presence of bovine rumen fluid.

To evaluate the potential use of the grids containing entrapped *α*-amylase to improve the performance of starch digestion in bovine rumen using corn grids, tests were conducted using pectin/PVA/amylase materials produced with 0.25% and 1.25% glutaraldehyde and using ruminal liquid containing free *α*-amylase.  Figure 6 shows that the profile of starch hydrolysis (corn grids) in terms of production of reducing sugar was quite different. The lower concentration of reducing sugar was observed in tests conducted with ruminal liquid containing free *α*-amylase. This result is in accordance with previous observations of low efficiency in starch hydrolysis in ruminal liquid and explains the presence of starch in the feces of the animal [3, 35]. The addition of exogenous *α*-amylase resulted in increased starch hydrolysis for both 0.25 and 1.25% glutaraldehyde grids. However, the profile of reducing sugar production was very different, as shown in Figure 6. Grids produced with 0.25% glutaraldehyde showed maximum production of reducing sugar after 2 h of incubation, after which the activity decreased to reach levels similar to those of the free *α*-amylase after 10 h of incubation. By contrast, the grids produced with 1.25% glutaraldehyde showed a consistent activity of *α*-amylase from 5 to 40 h of incubation.

A possible explanation for the best performance of grids obtained with 1.25% glutaraldehyde may be related to the higher number of linkages among the polymer chains in the blend network, which reinforced the grid structure and increased the resistance of the material to the action of microorganisms present in the ruminal liquid. As a consequence, the material is slowly degraded, and amylase remains active for a long period. Conversely, the material obtained with 0.25% glutaraldehyde presented a low number of cross-linking points in the polymer network, which favored its degradation and release of amylase into the ruminal liquid.

## 4. Conclusions

A new source of pectin was explored in this study to produce films by blending it with PVA and *α*-amylase and using glutaraldehyde as a cross-linker. The study showed that variations in glutaraldehyde concentration resulted in materials with different morphological and mechanical properties. Although the concentration of glutaraldehyde did not directly affect the initial activity of *α*-amylase, it did interfere in the performance of materials in tests of repeated use.* In vitro* tests using artificial rumen showed that material with higher mechanical resistance in terms of TS performed best in the continuous hydrolysis of starch using corn grids, recommending it as a promising material for improving ruminal starch digestion.

## Figures and Tables

**Table 1:** Composition of the reactors in trials of starch hydrolysis in artificial bovine rumen (*in vitro*).

Reactor	Bovine rumen (mL)	^∗^Ruminal buffer solution (mL)	Corn grain (g)	Pectin/PVA/amylase (g)	
				0.25%	1.25%
0.25%	400	1600	80	20	0
1.25%	400	1600	80	0	20
Standard	400	1600	80	0	0

^∗^Ruminal buffer composition (g L^−1^): 10 g KH_2_PO_4_, 0.5 g MgSO_4_ 7 H_2_O, 0.5 g NaCl, 0.1 g CaCl_2_ 2 H_2_O, 0.5 g Urea, 15 g Na_2_CO_3_, and 1.0 g Na_2_S 9 H_2_O.

**Table 2:** Tensile strength (TS) and elongation of pectin/PVA/amylase films.

Pectin/PVA/amylase	Tensile strength (kgf)	Elongation (%)
0.25%	0.26 (±0.015)^c^	41.44 (±0.96)^a^
0.50%	0.59 (±0.020)^b^	35.36 (±0.24)^b^
0.75%	0.52 (±0.030)^b^	41.08 (±1.58)^a^
1.00%	0.55 (±0.050)^b^	26.33 (±2.00)^c^
1.25%	0.69 (±0.050)^a^	24.22 (±1.32)^c^

^∗^Different letters in the same column indicate statistically different variations (*p* < 0.05). Values are the means of three independent measurements (M ± SD).

**Table 3:** Activity of *α*-amylase (U) in pectin/PVA/amylase materials as function of incubation period. Values are M ± SD from triplicates.

Time (h)	Grids				
	0.25%	0.50%	0.75%	1.0%	1.25%
2	13.50 (±1.4)	14.4 (±0.21)	11.6 (±0.503)	13.9 (±0.57)	14.28 (±0.24)
4	3.20 (±0.16)	1.83 (±0.24)	2.670 (±0.022)	2.66 (±0.393)	1.80 (±0.187)
6	0.28 (±0.03)	0.194 (±0.03)	0.247 (±0.048)	0.40 (±0.002)	0.24 (±0.02)
8	0.24 (±0.05)	0.14 (±0.023)	0.072 (±0.002)	0.06 (±0.001)	—
10	0.24 (±0.02)	0.10 (±0.013)	—	—	—
12	0.22 (±0.002)	—	—	—	—
14	0.16 (±0.01)	—	—	—	—
16	0.09 (±0.01)	—	—	—	—
18	0.13 (±0.02)	—	—	—	—
20	0.13 (±0.02)	—	—	—	—
22	0.10 (±0.04)	—	—	—	—
24	0.07 (±0.01)	—	—	—	—
